# Supplementary figures and images for: Large-scale analysis of SARS-CoV-2 spike-glycoprotein mutants demonstrates the need for continuous screening of virus isolates
Source: PLoS One. 2021 Sep 27;16(9):e0249254. doi: 10.1371/journal.pone.0249254 (PMC8475993; doi:10.1371/journal.pone.0249254)

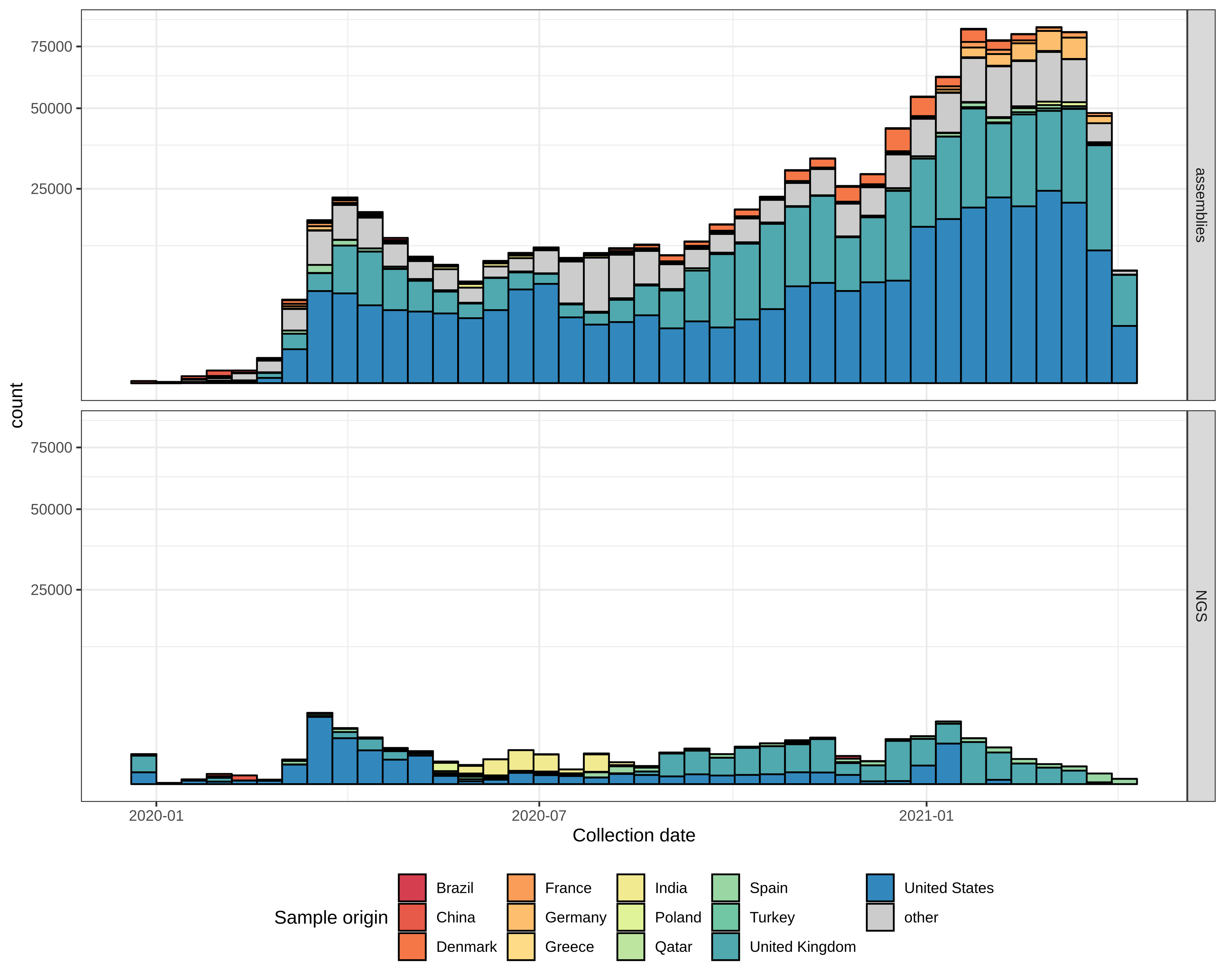

Supplement: S1 Fig — The histogram shows the number of SARS-CoV-2 assembly sequences deposited at GISAID and NGS data deposited at SRA. Color coding indicates the sample origin. Countries summarized as “other” include: Afghanistan, Albania, Algeria, Andorra, Angola, Antigua and Barbuda, Argentina, Armenia, Aruba, Australia, Austria, Azerbaijan, Bahamas, Bahrain, Bangladesh, Barbados, Belarus, Belgium, Belize, Benin, Bermuda, Bolivia, Plurinational State of, Bonaire, Sint Eustatius and Saba, Bosnia and Herzegovina, Botswana, Brunei Darussalam, Bulgaria, Burkina Faso, Cambodia, Cameroon, Canada, Cayman Islands, Chile, Colombia, Comoros, Congo, Costa Rica, Croatia, Cuba, Cyprus, Czechia, Dominican Republic, Ecuador, Egypt, El Salvador, Equatorial Guinea, Estonia, Eswatini, Ethiopia, Faroe Islands, Finland, French Guiana, French Polynesia, Gabon, Gambia, Georgia, Ghana, Gibraltar, Guam, Guatemala, Guinea, Hong Kong, Hungary, Iceland, Indonesia, Iran, Islamic Republic of, Iraq, Ireland, Israel, Italy, Jamaica, Japan, Jordan, Kazakhstan, Kenya, Kuwait, Latvia, Lebanon, Lesotho, Liechtenstein, Lithuania, Luxembourg, Madagascar, Malawi, Malaysia, Mali, Malta, Martinique, Mauritius, Mexico, Moldova, Republic of, Monaco, Mongolia, Montenegro, Morocco, Mozambique, Myanmar, Nepal, Netherlands, New Zealand, Nigeria, North Macedonia, Northern Mariana Islands, Norway, Oman, Pakistan, Palestine, State of, Panama, Papua New Guinea, Paraguay, Peru, Philippines, Portugal, Reunion, Romania, Russian Federation, Rwanda, Saint Barthelemy, Saint Kitts and Nevis, Saint Lucia, Saint Martin (French part), Saint Vincent and the Grenadines, Saudi Arabia, Senegal, Serbia, Sierra Leone, Singapore, Slovakia, Slovenia, South Africa, Sri Lanka, Suriname, Sweden, Switzerland, Taiwan, Province of China, Thailand, Togo, Trinidad and Tobago, Tunisia, Uganda, Ukraine, United Arab Emirates, unknown, Uruguay, Uzbekistan, Venezuela, Bolivarian Republic of, Vietnam, Virgin Islands, British, Zambia, Zimbabwe and unknown. (TIF) [file pone.0249254.s001.tif]
